# Lysophosphatidic Acid Receptors: Biochemical and Clinical Implications in Different Diseases

**DOI:** 10.7150/jca.41841

**Published:** 2020-03-15

**Authors:** Hongjiao Xiang, Yifei Lu, Mingmei Shao, Tao Wu

**Affiliations:** Center of Chinese Medical Therapy and Systems Biology, Institute of Interdisciplinary Integrative Medicine Research, Shanghai University of Traditional Chinese Medicine, Shanghai 201203, China

**Keywords:** lysophosphatidic acid, LPAR, structure, physiology and pathology, cancer

## Abstract

Lysophosphatidic acid (LPA, 1-acyl-2-hemolytic-sn-glycerol-3-phosphate) extracted from membrane phospholipid is a kind of simple bioactive glycophospholipid, which has many biological functions such as stimulating cell multiplication, cytoskeleton recombination, cell survival, drug-fast, synthesis of DNA and ion transport. Current studies have shown that six G-coupled protein receptors (LPAR1-6) can be activated by LPA. They stimulate a variety of signal transduction pathways through heterotrimeric G-proteins (such as Gα12/13, Gαq/11, Gαi/o and GαS). LPA and its receptors play vital roles in cancers, nervous system diseases, cardiovascular diseases, liver diseases, metabolic diseases, etc. In this article, we discussed the structure of LPA receptors and elucidated their functions in various diseases, in order to better understand them and point out new therapeutic schemes for them.

## Introduction

Lysophosphatidic acid (LPA, 1-acyl-2-hemolytic-sn-glycerin-3-phosphate), a small glycerophosphatidic acid, widely exists in human body. It has many different biological functions, such as promoting cell growth, differentiation, movement, survival and cytoskeleton morphological change 1. LPA combines with a variety of known G-protein coupled receptors (GPCRs) to perform a wide range of biological functions.

The six lysophosphatidic acid receptors (LPAR) currently known can be subdivided according to their homology. LPA_1-3_ receptor belongs to the endothelium differentiation gene (EDG) receptor, which has 50-57% amino acid identity with each other, while LPA_4-6_ receptor, which is a non EDG receptor with a long phylogenetic distance, has 35-55% amino acid identity with each other 2,3. Since the LPA receptors currently known are all G-protein coupled receptor families, they are all rhodopsin-like and have seven transmembrane domains, three extracellular loops (extracellular loop-ECL1, ECL2, ECL3) and three intracellular loops (intracellular loop-ICL1, ICL2 , ICL3). It also has an N-terminus within the cell and a C-terminus outside the cell 4. At least two Gα subunits (Gα12/13, Gαq/11, Gαi/o and GαS) are used by the LPA receptors to signal, thereby activating different downstream pathways, and under different environments and cell types produce different results 5. Numerous studies have shown that the role of LPA and its receptors is crucial in neurological diseases, tumors, metabolic diseases, liver diseases and cardiovascular diseases 6,7. In recent years, the role of LPA and its receptors in diseases has been paid more and more attention, and research into their mechanisms to find new disease treatment programs has become the focus of current research.

In this review, we hope to provide some new ideas and directions for the prevention and treatment of related diseases by summarizing the different functions of LPA receptors in different diseases.

## Structure of lysophosphatidic acid receptor

### LPAR1

**Figure 1:**
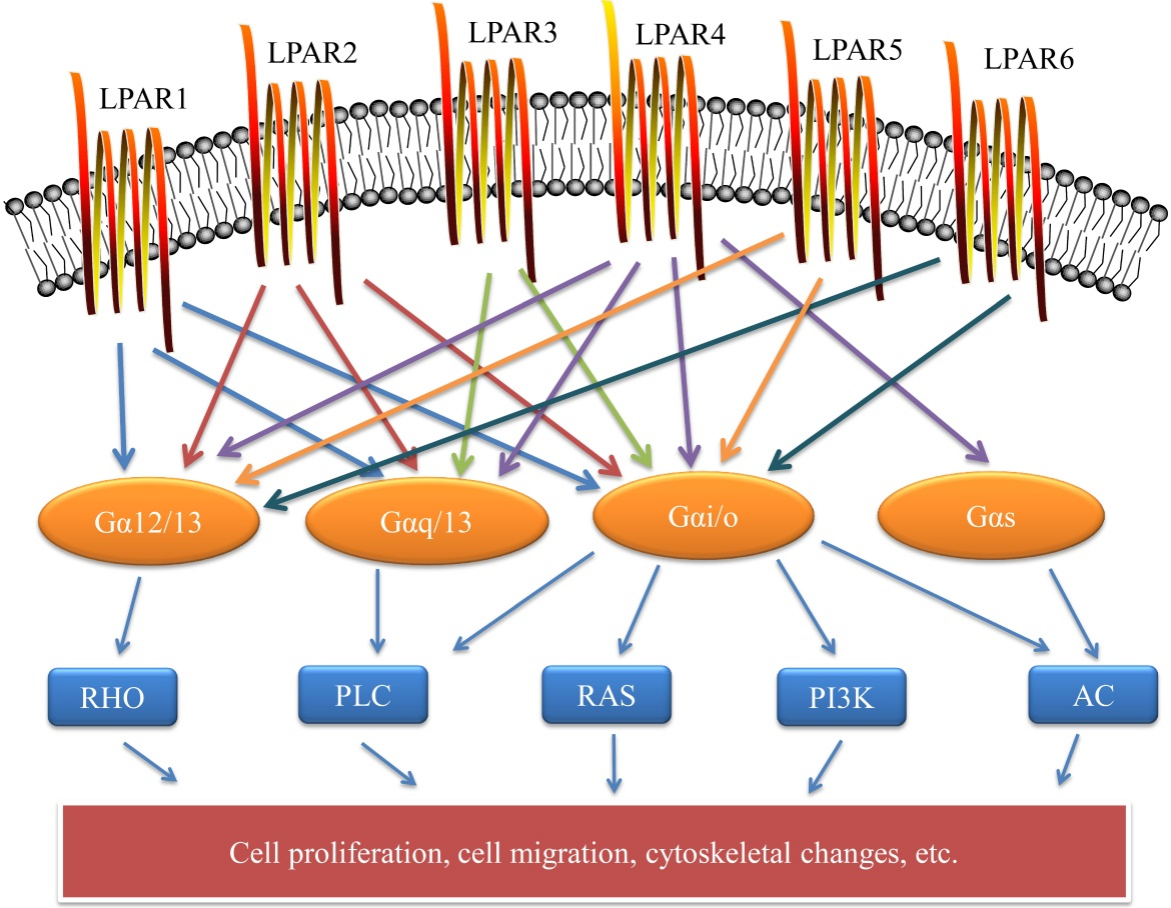
** Cell surface LPA receptors and their downstream signaling pathways.** LPA signaling is mediated through six known G protein-coupled receptors. The LPA receptor is a 7-TM structure that is coupled to at least two G protein family members to activate downstream pathways that mediate a variety of cellular responses. These include cell proliferation, cell migration, and cytoskeletal changes. LPAR: lysophosphatidic acid receptor; RHO: rho protein; PLC: phospholipase C; RAS: ras protein; PI3K: phosphatidylinositol 3-kinase; AC: adenylyl cyclase.

LPAR1, the earliest LPA receptor, was found in the neuroproliferative ventricular zone (VZ), superficial marginal zone, and meninges in a brain study in 1996 and identified as a receptor mediating LPA action 8. LPA_1_ is widely expressed in various tissues and organs of human body, in which the mRNA levels in brain, heart, colon, small intestine and placenta are higher, but relatively lower in other organs and tissues 9. LPAR1, a 41 kDa protein, consists of 364 amino acids. Its human chromosome gene is located on chromosome 9 (9q31.3), and has 7 transmembrane domains like other LPA receptors (Figure 1) 10. Three extracellular loops and three intracellular loops were formed by patterning 7 times on the plasma membrane. The transmembrane span III (TMIII) and transmembrane spanning V (TMV) and transmembrane spanning VII (TMVII) linkages to ECL1 and ECL2 are key regions for LPA binding to LPA_1_. The activation and desensitization of intracellular signals is associated with the ICL2 region, while the smallest ICL1 allows the cytoplasmic organelle to correctly process the receptor and express it on the cell surface. Among several intracellular and extracellular loops, ICL3, which has 33 amino acids, is the largest. It is a key region for LPA_1_ signal transduction and attenuation 11. It was also found that LPA1 preferred to receive ligands in extracellular environment 12. The receptor activates downstream pathways such as Akt, rho, mitogen-activated protein kinase (MAPK) and phospholipase C (PLC) by coupling with Gαi/o, Gαq/11 and Gα12/13. LPA mediates a great diversity of functions through LPA_1_ coupled to the G protein, including cell survival, cell proliferation, cell adhesion, cell migration, cytoskeletal changes, Ca^2+^ mobilization, immune function, and myelination 5. Such as promoting astrocyte proliferation and neuronal differentiation, proliferation of oligodendrocytes and smooth muscle cells, migration and anti-apoptosis of Schwann cells, mineralization and osteogenic transformation of valve interstitial cell (VIC) 13,14. Approximately 50% of LPA_1_-deficient mice presented neonatal mortality, which may be due to the lack of olfactory agent detection or olfactory processing in these mice, resulting in their inability to locate nipples for breast-feeding. These mice also showed changes characterized by nose shrinkage, eye spacing enlargement, cerebral cortex development changes and weight loss. Besides, some mice also experienced frontal cranial hemorrhage 5.

### LPAR2

LPA_2_ was identified from a gene library homology search of orphan GPCR genes, which has approximately 60% amino acid similarity to LPA_1_ 15. LPAR2 is a receptor encoding 348 amino acids with a molecular weight of about 39 kDa. The human gene is located on chromosome 9 (19p12) 9. The coding region of the mouse LPA_2_ gene containing three exons is located in exons 2 and 3 16. LPA_2_ receptor mRNA expression in kidney, uterus, testis and leukocytes was higher than that in thymus, pancreas and spleen. LPA_2_ is the same as LPA_1_, is also coupled with Gαi/o, Gαq/11 and Gα12/13 in the heterotrimeric G protein family. In turn, through Ras, MAPK, phosphatidylinositol 3-kinase (PI3K), Rac, PLC, diacylglycerol (DG) and Rho and other downstream molecules transmit signals that mediate cell survival and cell migration 5,9. The PSD-95/DlgA/ZO-1 (PDZ) binding motif is located at the carboxy terminus of the LPA_2_ receptor and interacts with a variety of PDZ scaffold proteins. These scaffold proteins include Na^+^/H^+^ exchange regulatory factor-1 (NHERF-1), Na^+^/H^+^ exchange regulatory factor-2 (NHERF-2), inverted orientation-2 (MAGI-2), inverted orientation-3 (MAGI-3), neurabin, PDZ-RhoGEF (PRG) and leukemia-associated Rho GEF (LARG) proteins. The interaction between NHERF-2 and LPA_2_ enhanced LPA mediated cell proliferation and cell migration, while the interaction between MAGI-3 and LPA_2_ resulted in the opposite effect of NHERF-2. MAGI-3 had a negative regulatory effect on LPA_2_ mediated cell function 17. Studies have shown that spatial organization of PDZ motif-mediated LPA_2_ receptor macromolecular complex assembly mediates LPA gradient sensing in fibroblasts 18. LPA mediates anti-apoptosis of cells through LPA_2_ receptor, and the damage repair effect of LPA_2_ receptor on DNA can protect cells from radiation to some extent 19. There was no apparent phenotypic abnormality in LPA_2_-deficient mice, but in LPA_1_ and LPA_2_-deficient mice, the incidence of frontal lobe hematoma increased during perinatal period 9.

### LPAR3

LPA_3_ was discovered similarly to LPA_2_ and cloned by a cloning method based on degenerate polymerase chain reaction 20. LPAR3, a human chromosomal gene, is located at 1p22.3-p31.1 and consists of 353 amino acids, with a molecular weight of approximately 40kDa. LPA_3_, located on the X chromosome, is similar to LPA_1_ and LPA_2_ in that it has about 50% amino acids 9,21. LPAR3 is coupled to Gαi/o and Gαq of the heterotrimeric G protein, mediating PLC activation, Ca^2+^ mobilization, inhibition and activation of adenylate cyclase (AC), and protein kinase activation that mediates mitogen activation 22. The mRNA level of LPAR3 is higher in human heart, lung, pancreas, brain, testis, prostate and ovary. However, in mice, the mRNA levels of LPAR3 in testis, kidney, thymus, small intestine, lung, stomach and brain are higher 9. Under the action of LPA_3_, immature mouse dendritic cells can be chemotaxis to LPA 23. The LPA_3_ receptor can also participate in the mobilization and recruitment of smooth muscle progenitor cells (SPC) and mediate the development of central post-stroke pain (CPSP) along with the LPA_1_ receptor 14,24. In contrast to mice lacking LPA_1_ and LPA_2_, the absence of LPA_3_ resulted in delayed embryo implantation and changes in embryo spacing, which dramatically reduces embryo crowding and litter size. The effect of LPA_3_ on embryo implantation is related to cyclooxygenase-2 (COX-2) and its derived prostacyclin (PGI2), as the lack of COX-2 leads to reproductive failure in multiple female mice 25,26. The lack of LPA_3_ receptor alone did not show a definite change in male reproduction, but the LPA_1_ receptor, LPA_2_ receptor and LPA_3_ receptor combined with defective male mice showed decreased germ cell survival and increased azoospermia prevalence 26. The above studies suggest that LPA_3_ is closely related to reproduction.

### LPAR4

The LPA_4_ receptor discovered by ligand screening differs from the amino acid sequence of LPA_1-3_. Its amino acid similarity with LPAR1-3 is only 20-24%. LPA_4_ is structurally distinct from previously identified EDG family receptors (LPA_1_, LPA_2_, LPA_3_) and more similar to P2Y purinergic receptors 27. In humans, the 370 amino acid LPA_4_ gene is located in the q13-q21.1 region of the X chromosome and contains a 1113 bp intron-free open reading frame with a molecular weight of about 40 kDa 9,27. LPAR4 mRNA levels are higher in mouse skin, heart, ovary, thymus, developing brain and embryonic fibroblasts, and levels in the ovary are more pronounced 7,9. LPA_4_ binds primarily to Gαq, Gαi, Gα12/13 and Gαs to activate downstream pathways 28. LPA_4_ induces intracellular cyclic adenosine monophosphate (cAMP) accumulation by Gαs 27, cell aggregation and adhesion by N-cadherin 29, as well as neurite contraction and stress fiber formation by activating the Rho/ROCK pathway 30. Other studies have shown that it can affect the differentiation of immortalized hippocampal progenitor cells 31. In contrast to the LPA_1_ receptor, LPA-mediated migration and invasion of the cell are inhibited by the LPA_4_ receptor; for example, studies have found that continued progression of rat neuroblastoma and human colon cancer can be inhibited by LPA_4_ 32,33. Different degrees of subcutaneous hemorrhage and vasodilation, impaired vascular endothelial cell wall cell coverage, and lymphatic dysplasia were observed in LPA_4_-deficient mouse embryos 34. It indicates that LPA4 receptor can promote angiogenesis and vascular development. In addition, the regulatory factor Yes-associated protein (YAP) and the transcriptional coactivator with PDZ-binding motif (TAZ) can also be activated by LPA_4_/LPA_6_ via the Gα12/Gα13 signaling pathway 35. YAP and TAZ can accelerate the progression of cancer by promoting the proliferation and migration of cancer cells, such as accelerating liver cancer, bladder cancer and lung cancer 36-38. Also, the volume, number and thickness of trabeculae increased in adult LPA_4_^-/-^mice, contrary to those observed in LPA_1_^-/-^mice, suggesting that LPA_4_ has a negative regulatory effect on bone formation and can counteract LPA_1_-induced bone formation 39.

### LPAR5

As the fifth reported LPA receptor, LPA_5_ was identified as a member of the LPA receptor by using reverse transcription in an unbiased screening method. LPAR5 shares 35% homology with LPAR4, but with LPAR1-3 only 22% homology 40. The molecular weight of LPAR5 consisting of 372 amino acids is about 41 kDa. The human gene is located at 12p13.31. Large amounts of LPAR5 are expressed in the spleen, small intestine, and colon, but are relatively low in most other tissues 7,40,41. LPAR5 has multiple endogenous ligands including LPA, geranylgeranyl diphosphate (GGPP), farnesyl monophosphate, farnesyl pyrophosphate, N-arachidonoyl Glycine alkyl glycerophosphate and cyclic phosphatidic acid 41,42. Stress fiber formation and neurite contraction are associated with the LPA-LPA_5_-Gα12/Gα13 pathway, whereas increased intracellular calcium levels and cAMP accumulation are associated with the LPA-LPA_5_-Gαq pathway 7,9,40. LPA binds to LPA_5_ expressed on mouse and human CD8^+^ T cells, inhibited T cell antigen receptor (TCR)-induced intracellular calcium mobilization, extracellular regulated protein kinases (ERK) activation and Nur77 expression, thereby reduced granular exocytosis and cytotoxicity 43. LPA_5_ is also expressed in the spinal cord and dorsal root ganglia (DRG) and can promote the signaling of extensive pain in the spinal cord 42. In intestinal epithelium, LPA_5_ is the primary LPA receptor regulating Na^+^/H^+^ Exchanger 3 (NHE3). It co-expressed with NHERF-2, induces Na^+^-dependent water absorption, and recruits NHE3 to intestinal microvilli 41,44. Studies have shown that the transmission of pruritus signals caused by LPA is mainly transmitted through LPA_5_, PLD; transient receptor potential vanilloid 1 (TRPV1) and transient receptor potential ankyrin 1 (TRPA1) 45. Whether LPA_5_ deficiency leads to disease-related changes is currently unclear.

### LPAR6

As the sixth prion-coupling receptor for LPA; LPAR6, formerly known as P2Y5, was recognized as an LPA receptor in 2008 and is thought to be closely related to hair growth. The location of LPAR6 on chromosome is 13 (13q14), which encodes 344 amino acids with a molecular weight of about 39 kDa 5. Like LPA_4_, it belongs to the P2Y receptor family. The ligand-binding pattern of LPA_6_ is quite different from that of LPA_1_, and the ligand binding pocket of LPA_6_ is transverse to the membrane opening, and the acyl chain of the lipid used for crystallization is incorporated therein 46. LPAR6 binds to Gαi and Gα12/13 and activates downstream pathways for multiple functions. Activation of the Gα12/13 family induces expression of SRE regulatory genes by activating Rho and ROCK 47. LPA_6_-mediated Rho-dependent morphological changes and cAMP accumulation were detected by using chimeric Gα13 protein 48. LPA_6_ mostly binds to 2-acyl-LPA instead of l-acyl-LPA. When the promiscuous Gα protein is co-expressed with LPA_6_, LPA activates LPA_6_ to increase intracellular Ca^2+^ and reduce cAMP and ERK1/2 activation which stimulated by forskolin 9,47. As already mentioned, the transcriptional regulators YAP and TAZ can be activated by the LPA-LPA_4_/LPA_6_-Gα12/Gα13 signaling pathway, and both YAP and TAZ are involved in tumor progression 35. Moreover, another article mentions the binding of LPA to the LPA_6_ receptor to regulate vascular permeability 49. LPA_6_ also regulates the formation of hair follicles, and the loss of LPA_6_ leads to congenital alopecia 46,50. Research on the role and mechanism of LPAR6 in different systems is still relatively rare, which may be a future research direction.

## The role of LPAR in diseases

### LPAR and cancer

**Figure 2:**
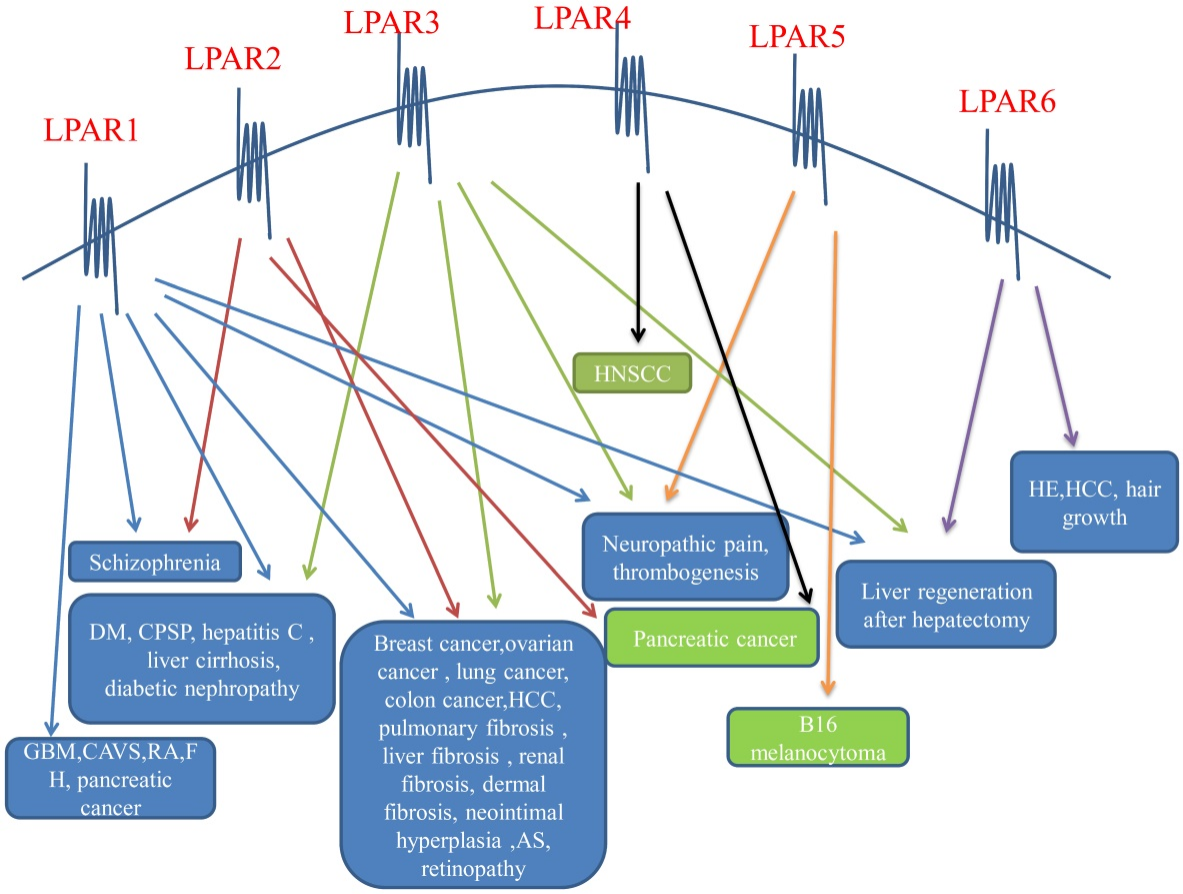
** LPA receptors on the cell surface and their effects on diseases.** The six LPA receptors have different effects on diseases of different systems, and both promote and inhibit the disease. The blue text box in the above figure indicates that the LPA receptors promotes it, and the green text box indicates that the LPA receptors inhibits it. LPAR: lysophosphatidic acid receptor; DM: diabetes mellitus; CPSP: central post-stroke pain; GBM: glioblastoma multiforme; CAVS: calcified aortic stenosis; RA: rheumatoid arthritis; FH: fetal hydrocephalus; HCC: hepatocellular carcinoma; AS: atherosclerosis; HNSCC: headneck squamous cell carcinoma; HE: hepatic encephalopathy.

LPA induces cell proliferation, migration and survival. LPA affects cell morphology, mobility, chemotaxis and invasiveness by stimulating cell cycle, increasing cell viability, and promoting the production of interleukin (IL)-6, IL-8 and vascular endothelial growth factor (VEGF), interleukin (IL)-6, and IL-8. There is a lot of evidence that LPA plays an indispensable part in the occurrence and development of cancer because of its functions 5,51,52. The purpose of LPA in cancer is mainly achieved through its G protein coupled receptor. EDG receptors (LPA_1-3_) play a significant character in various cancers, but the character of non-EDG receptors (LPA_4_, LPA_5_, LPA_6_) in cancer is currently less studied. Different receptors of LPA receptors play different roles in various tumors to push the proliferation, migration, and anti-apoptosis of tumor cells and play a role in preventing the advancement of tumors (Figure 2).

#### LPAR and ovarian cancer

A large amount of LPA is present in the ascites of ovarian cancer. Overexpression of LPA_2_ and LPA_3_ in ovarian cancer cells stimulate proliferation and migration of ovarian cancer cells 53. The level of LPAR1 protein was significantly higher in lymph node metastasis and relapsed more in OSC tissues than in primary tumor lesions, and high levels of LPAR1 protein suggested poor prognosis 54. The LPA_2_ receptor induces the expression of VEGF by participating in the production of IL8 and induces the expression of cyclin D1 through transcriptional activation, thereby stimulating the growth of ovarian cancer and enhancing the invasiveness of ovarian cancer 51,52,55,56. The study also found that LPA stimulated COX-2 expression and cell movement through the LPA_2_-Gαi-Src-epidermal growth factor receptor (EGFR)-ERK signaling cascade in ovarian cancer cells 57. The expression of LPA_3_ receptor is mainly related to malignant cells. LPA_3_ selectively increases in ovarian cancer cells, and LPA with unsaturated fatty acyl chains is preferred in sn-LPA (common in ascites of ovarian cancer) 56. Yu et al. reported that the tumor volume and invasiveness of mice injected with SKOV-3 cells expressing LPA receptors increased, and the concentrations of IL-6, IL-8 and VEGF in mouse ascites and serum increased significantly. And tumor volume and invasiveness of LPA_2_ and LPA_3_ were significantly increased. HIF-1α is closely related to the occurrence and development of tumors 56. Ha et al. found that the LPA-LPAR-Gαi2 axis can induce pseudo hypoxic response through the Rac-NOXROS-HIF1α pathway, which ultimately leaded to metabolic reprogramming of ovarian cancer cells. This conclusion was verified by establishing two independent mouse models of ovarian cancer cell line xenograft (CDX) 58. PI3K/Akt pathway is critical in the process of ovarian cancer. LPAR1 plays a paramount role in the invasion, proliferation, migration, and development of ovarian serous cystadenocarcinoma (OSC) intratumoral heterogeneity (ITH) by regulating the activity of PI3K/AKT signaling pathway 54. When miR-15b was up-regulated, the proliferation of ovarian cancer cells and their apoptosis and senescence were prohibited by the LPAR3-PI3K/Akt pathway 59.

#### LPAR and breast cancer

In breast cancer (BC), the autotaxin (ATX)-LPA axis promotes BC cell invasion, proliferation and anti-apoptosis through high ATX expression and binding to LPA_1-3_, thereby brings about mammary gland inflammation and tumor formation. This is related to with activation of the LPA receptors by the ATX-LPA axis, resulting in downstream pathways PI3K/Akt, p38-PI3K and ERK/MAPK, wnt/β-catenin, estrogen receptor (ER), and cytokine activation 60. The metastasis of cancer cells mainly involves LPA_1_ receptors. Debio-0719 (LPA_1_ receptor inhibitor) can prohibit the metastasis of cancer cells to the lung and liver in 4T1 mouse breast cancer model, and Debio-0719 can also hinder the metastasis of cancer cells to the lung in MDA-MB-231T human breast cancer model. LPA_1_ siRNA does not alter primary tumor size, but also inhibits cancer cell metastasis 61. LPA_1_ receptor can also promote xenograft bone metastasis of breast cancer 62. All these indicate that metastasis of breast cancer is closely linked to LPA_1_ receptor. In the previous discussion of ovarian cancer, we mentioned that the effect of LPAR on the transcription factor hypoxia-inducible factor-1α (HIF-1α) is a factor in the development of ovarian cancer. Li et al. examined the tissue samples of 156 BC patients and found that the expression of LPA_2_ in BC tissues was higher than that in adjacent tissues, and the expression of LPA_2_ was more in postmenopausal women. This indicates that the overexpression of LPA_2_ is closely related to the canceration of postmenopausal BC. In vitro experiments have also shown that LPA_2_ is positively correlated with HIF-1α expression, and can also promote the proliferation, migration and invasion of BC cells 63. Liu et al. found that transgenic mice overexpressing ATX and LPAR1-3 had an increased risk of breast cancer, and LPAR3 transgenic mice had the highest metastasis rate 62. Popnikolov and others confirmed that 87 patients with invasive human breast cancer were more prone to lymph node metastasis by immunohistochemical analysis. This suggests that LPA mediates tumor aggressiveness primarily through the LPA_3_ receptor 64. In addition, LPA_3_ can contribute to the growth of triple-negative breast cancer cell lines and is of great significance for the early diagnosis of triple-negative breast cancer 65,66. Tao et al. tested 98 clinical BC and adjacent tissues and found that the expression of LPAR6 in BC was much lower than that in adjacent tissues, and the results were consistent with those obtained from the database. Knockdown of LPAR6 in cell experiments enhanced tumor cell proliferation and invasion. It is suggested that LPAR6 may play a role in inhibiting tumor development in BC 67.

#### LPAR and colon cancer

In colon cancer, LPA makes human colon cancer cell DLD1 more susceptible to metastasis by binding to LPA_1_. LPA enhances colon cancer cell proliferation and angiogenesis factor production via LPA_2_ 68. Earlier studies found that LPA_2_ receptors were important receptors for LPA in the colon and mediated mitogenic signals in human colon cancer cells 69. Lin et al. found that in LPA_2_^-/-^mice, mucosal damage in the colon was reduced and tumor growth was suppressed 70. LPA_2_ and LPA_3_ are targets of LPA-induced proliferation of HCT116 and LS174T colon cancer cells by specific RNA interference (RNAi), and LPA_2_ and LPA_3_ promote the proliferation of colon cancer cells through classical protein kinase C (cPKC)-mediated activation of the β-catenin pathway 71. LPA_2_ receptor signaling regulates the function of colon cancer cells and is also associated with membrane-associated guanylate kinases, including MAGI-3 and NHERF-2. NHERF-2 competes with MAGI-3 for binding to LPA_2_, which has the opposite effect on the function of the LPA_2_ receptor to regulate colon cancer cells. The migration and invasion of colon cancer cells was inhibited when LPA_2_ binds to MAGI-3, and the opposite effect to NHERF-2 17. Beyond that, it was found that LPA_4_ and LPA_6_ can inhibit the locomotor activity of colon cancer cells 33.

#### LPAR and liver cancer

If factors such as hepatitis are not removed in time, the liver will continue to be damaged, and fibrosis will intensify, which may develop into cirrhosis and liver cancer. Hepatocellular carcinoma (HCC) is a type of primary hepatic carcinoma, accounting for 90% of primary liver cancer. In HCC tissues, LPA expression was higher and LPA receptors were also highly expressed. In liver cancer, studies have found that the expression of ATX and LPA_1_ receptors in HCC was higher than that in normal tissues. LPA_1_ receptor inhibition and inhibition of phosphoinositide 3-kinase (PI3K)/Akt and protein kinase Cd (PKCd)/p38-MAPK pathways all result in decreased MMP-9 activity and invasiveness of HCC. This demonstrates that LPA enhances MMP-9 expression and HCC invasion through LPA_1_ receptor and synergistic activation of the PI3K and p38MPAK signaling cascades 4,72,73. The migration ability of SKHep1 cells can be enhanced by LPA_3_-Gαi-ERK-MAPK signaling pathway, indicating that LPAR3 can promote the development of liver cancer 74. Okabe et al. also suggested that LPAR3 was related to the migration of liver cancer cells 75. Mazzocca et al. confirmed in vitro experiments that LPAR6 promoted the proliferation and tumorigenic phenotype of HCC cells, and established a relevant model to prove that LPAR6 promotes tumor growth, and the higher the expression level of LPAR6 in tumor tissues of liver cancer patients, the worse the prognosis of patients. Abnormally expressed LPAR6 can activate the protooncogene pim-3 via the signal transducers and activators of transcription 3 (STAT3) binding site, thereby maintaining the tumor proliferative capacity and tumorigenic capacity of HCC 76. LPAR1/3/6 mRNA expression was more elevated than non-tumor liver tissue, and LPA_6_ mRNA expression was highest. Regarding the role of LPA and its receptors in hepatocellular carcinoma, the researchers suggest that on the one hand, LPA produced by other pathways promotes the development of HCC. On the other hand, HCC produces LPA and can continue to promote HCC progress in combination with LPAR. For example, the growth and progression of HCC are maintained along with the autocrine loop produced by LPAR6 in tumor cells 77,78. These results indicate that the function of LPA and its receptors in the development of HCC cannot be ignored. In addition, Enooku et al. analyzed liver cancer tissues and adjacent tissues of 58 patients with HCC and found that the high expression of LPAR2 and LPAR6 often indicated that the tumor has a higher degree of malignancy 79.

#### LPAR and pancreatic cancer

In pancreatic cancer, the progress of PANC-1 cells can be promoted by LPA_1_ and LPA_3_, suggesting that LPAR1 and LPAR3 contribute to the movement and development of pancreatic cancer cells 80, while LPA actively inhibits the action of pancreatic cancer cells through LPAR2-Gα12/13-Rho signal transduction pathway 81. Several other studies have also shown that LPAR1 can promote the metastasis and invasion of pancreatic cancer cells, while LPAR2 has the opposite effect 81,82. Komachi et al. used oral administration of Ki16198 (specific inhibitors of LPAR1 and LPAR3) in nude mice modeled with pancreatic cancer cells, which inhibited tumor growth and reduced invasion and metastasis of other organs 83. Yang et al. proposed that YAP promoted the migration and invasion of pancreatic cancer cells by up-regulating LPAR3 in cells 84. Furthermore, by using shRNA to establish knockdown models-PANC-sh4, PANC-sh5, PNAC-sh6, LPA_4_ was found to inhibit the development of lung cancer, while LPA_5-6_ promoted pancreatic cancer 65.

#### LPAR and glioblastoma

Glioblastoma (GBM) is one of the most aggressive brain tumors. Earlier research discovered that LPA_1_ was highly expressed in the brain and participated in various activities of the nervous system 5. Recently, LPA_1_ receptor was discovered related to the proliferation and migration of GBM. In three GBM cell lines, LPA induces PKCα activation by LPA_1_, leading to nuclear translocation of kinases, increasing cell number and increasing cell viability 85,86.

#### LPAR and melanoma

LPAR1, LPAR2 and LPAR5 play essential roles in the invasion and metastasis of melanoma. Host LPA_1_ and LPA_5_ receptors promote B16F10 melanoma cell-derived lung metastasis. The expression of LPA_2_ in tumor cells promotes invasion, but LPA_5_ inhibits invasion. Jongsma et al. also proposed that LPA_5_ receptors inhibited melanoma cell migration and were related to downstream cAMP 87. It was worth noting that LPA_5_ receptor on cytotoxic CD8^ +^ T cells acts contrary to LPA_5_ receptor in tumor cells. Its activation inhibits the activation and proliferation of T cells, thus facilitating the escape of host immunity 72. In another study, by using LPAR1/3 antagonists, it was suggested that LPAR3 was related to the activity of B16F10 metastatic melanoma cells and may be an important target for the treatment of melanoma 88.

#### LPAR and lung cancer

In A549 lung tumor cells, LPA inhibits P53 activity via LPA_1-3_ receptor, reduces p53-dependent transcription, promotes loss of p53 protein, and protects tumor cells from actinomycin D-induced apoptosis 89. It is suggested that the expression of LPA_1-3_ receptor contributes to the development of lung cancer. However, it was found that increased migration of rat lung tumor cells may be related to the loss of LPAR3 90. Yamada et al. proposed that mutations in the LPAR1 gene promoted the development of rat lung adenomas into lung adenocarcinomas 91. Magkrioti et al. also proposed that gene deletions of LPAR1 and nucleotide pyrophosphatase/phosphodiesterase 2 (ENPP2) slowed the progression of lung fibrosis and lung cancer 92.

In the above discussion, we can see that EDG receptors are immeasurable in the progress of cancer, and the role of the same LPA receptor in different cellular environments was not the same. Future research can focus on the study of anticancer drugs based on LPA receptors.

### LPAR and nervous system disease

Lysophosphatidic acid (LPA) is essential for brain development and nervous system function, signaling through six different G-protein coupled receptors (LPAR1-6). The role of LPA receptors in the nervous system is one of the earliest research findings. The LPA_1_ receptor was first discovered in the VZ, and the LPA_1_ receptor was most widely distributed in the nervous system 8.

#### LPAR and neuropathic pain

Through pharmacological and genetic studies, it was found that in mice lacking the LPA_1_ receptor, due to the inability to activate the Rho-ROCK pathway, abnormal mechanical pain, thermal hyperalgesia and demyelination, up-regulation of PKCγ and Caα2δ1 expression was inhibited, and the same phenomenon occurred after intrathecal injection of BoTXC3 into peripheral nerves 93. In a novel CPSP model developed by photochemically induced thrombosis (PIT) and tissue plasminogen activator, LPA_1/3_ antagonist Ki-16425 treatment can reverse established heat or mechanical hyperalgesia, blocking established CPSP. This also indicates that LPAR1 and LPAR3 are involved in neuropathic pain 24. Microglia are involved in the physiological and pathological processes of the nervous system and are permanent immune cells in the central nervous system (CNS). LPA may activate macrophages/microglia via LPAR1 and LPAR3 and promote self-amplification of LPA, increasing microglial migration and pro-inflammatory phenotype through the LPAR5/protein kinase D (PKD) axis. Activation and migration of microglia are associated with demyelination in the spinal cord after injury, and microglia are more involved in the initiation of neuropathic pain (NP) 94,95. In the cuprizone (CPZ)-induced multiple sclerosis (MS) model, LPA_5_ signaling mediates pain allergy induced by A-delta fibers and demyelination produced by CPZ. At the same time, phosphorylation of cAMP response element binding protein may lead to LPA_5_-mediated hyperalgesia in peripheral nerve injury in mice. This indicates that LPA_5_ signaling is related to neuropathic pain mediated by multiple sclerosis 96. It was found that intravenous LPA and GGPP (LPA_5_ agonists) induced allodynia, but GGPP-induced allodynia did not show up in LPA_5_-KO mice, indicating pain signals in the spinal cord by LPA_5_ transfer 94-96.

#### LPAR and fetal hydrocephalus

There is a common neurological disease in the newborn, fetal hydrocephalus (FH), and its occurrence is closely related to LPA and its receptors. In a mouse model of intracranial hemorrhage, by exposing the mouse embryonic brain to blood or LPA, the LPA_1_ receptor, which is dependent on the expression of neural progenitor cells (NPC), is over-activated, resulting in disruption and thinning of the cortical layer, ultimately leading to FH. FH was also found to be staged, suggesting that LPA receptors modulators can be used in the short-term to improve hydrocephalus without affecting LPA-mediated normal cortical development 97. Park et al. demonstrated that LPA may be involved in the development of fetal hydrocephalus by regulating the expression of the downstream factor Yap 98. When LPA was used to induce the production of posthemorrhagic hydrocephalus (PHH) by LPAR1-5 single gene knockout mice, LPAR1 and LPAR3 were found to be the main receptors of LPA-induced PHH production. When Ki16425 (LPAR1/3 inhibitor) was used in mice without gene knockout, the probability and severity of PHH production were reduced. This indicates that LPAR1 and LPAR3 may be key receptors for fetal hydrocephalus 99.

#### LPAR and hepatic encephalopathy

In hepatic encephalopathy mice, elevated serum ATX activates the LPA6-associated Gα12/13-Rho pathway in cerebral capillary vessel endothelial cells, resulting in enhanced blood-brain barrier (BBB) permeability and brain edema. The role of the LPA6 receptor in hepatic encephalopathy is suggested 100, 101. In addition, LPA and the tricyclic antidepressant amitriptyline (TCA) signaled LPAR1 to cut down P-glycoprotein transport in the BBB, thereby increasing drug delivery in blood-brain therapy 102.

#### LPAR and Alzheimer's disease

Alzheimer's disease (AD) is a chronic neurodegenerative disease distinguished by cognitive deterioration and behavioral abnormalities. LPA receptor-mediated Aβ accumulation, tau hyperphosphorylation and neuronal dysfunction are associated with AD. At the same time, traumatic brain injury, metabolic syndrome and chronic hypoperfusion are all mediated by LPA receptors and can be further developed into AD 103.

#### LPAR and retinopathy

The retina was an extension of the nervous system, and studies have shown that LPA was associated with retinopathy. In adult rats, the expression of LPA1 and LPA2 in retinal ganglion cells increased significantly after retinal ischemia, and LPA1 mediated retinal ganglion cell death in retinopathy of premature infants. In contrast, LPAR1-3 expressed by retinal pigment epithelial cells can promote retinal healing. It suggests that LPA has neuroprotective effects on the retina by binding to different LPA receptors on different cells, or has a neurodegenerative effect 104. LPAR1 and LPAR2 were also associated with schizophrenia 100,104.

In summary, considering the role of LPA in the nervous system, we can develop corresponding drugs to treat nervous system diseases by studying the mechanism of LPA and its receptors in the nervous system.

### LPAR and cardiovascular system disease

With lifestyle changes and an aging population, cardiovascular disease (CVD) has become a major threat to the health. In China, the prevalence of CVD is high, with about 290 million people affected by CVD, and the number is still growing 105. LPA and its receptors are critical for the development of cardiovascular disease. First, as mentioned earlier, the LPA4 signaling pathway in the vascular endothelium was essential for vascular development. Furthermore, the process of atherosclerosis and thrombosis was also associated with LPA and its receptors 34.

#### LPAR and atherosclerosis

When blood vessels are damaged, phenotypic regulation of vascular smooth muscle cells (SMCs) (including dedifferentiation, proliferation, and migration) and CXC motif ligand 12 (CXCL12)-dependent SPCs mobilization, as well as promotion of intimal hyperplasia, occur. These are the critical causes of atherosclerosis and stenosis. The ability of LPA_1_ and LPA_2_-deficient mouse vascular SMC to migrate is reduced, and the intimal hyperplasia caused by vascular injury is reduced, but only LPA_1_-deficient mice may have an effect of enhancing vascular injury. This may be related to the increased migration of vascular smooth muscle cells caused by concurrent LPAR3 compensatory upregulation. It was worth noting that although LPA was associated with blood pressure regulation, it was not associated with LPAR1 and LPAR2 106. The use of Ki16425 (LPA_1_ and LPA_3_ antagonists) after vascular injury significantly reduced neointimal expression of CXCL12 and hypoxia-inducible factor (HIF)-1α, mobilization of CXCL12-dependent SPCs, and also inhibited neointimal hyperplasia. These indicate that LPA_1_, LPA_2_ and LPA_3_ mediate the formation of neointimal after vascular injury 14. In addition, the long-term use of Ki16425 can also inhibit the recruitment of monocytes that can lead to atherosclerosis. Studies have shown that LPA promotes the adhesion of monocytes induced by CXCL1 through the release of endothelial cell CXCL1 mediated by LPA_1_ and LPA_3_ receptors 107. The occurrence of atherosclerosis was also associated with inflammation. In recent studies, it was found that in LDLr^-/-^mice, Ki16425 induced a systemic anti-inflammatory response by inhibiting CCL2-CCR2 signaling. This enhanced the anti-inflammatory innate and adaptive immune response and lowered plasma cholesterol levels, ultimately damaging the progression of atherosclerosis 108. In summary, LPA_1-3_ was a critical aim for the cure of atherosclerosis and neointimal formation after stent implantation.

#### LPAR and thrombogenesis

Thrombogenesis is another risk factor for cardiovascular disease. In one aspect, LPA mediates platelet activation by stimulating LPAR1 and LPAR3, and LPA was a crucial thrombogenic component of the plaque lipid core. Selective antagonists of LPAR1 and LPAR3 dioctylphosphatidic acid [PA (8:0)] and dioctylglycerol pyrophosphate [DGPP (8:0)] can selectively suppress induction by LPA and mox-LDL Changes in platelet shape (inhibition of platelet activation by LPA), thereby reducing the formation of intravascular thrombus 109. On the other hand, plaque rupture caused by the further development of atherosclerotic plaques can lead to acute thrombotic occlusion of the arterial lumen, and there was a risk of cardiovascular diseases such as myocardial infarction and stroke. Studies have shown that after rupture of atherosclerotic plaques, LPA exposed from lipid nucleus induces platelet shape changes by binding to LPA_5_ and synergizes with adenosine diphosphate (ADP) to stimulate platelet aggregation and thrombosis 110. In another study on thrombosis, it was also suggested that LPA can enhance the assembly of fibrin and thus promote thrombosis 111.

#### LPAR and calcified aortic stenosis

Calcified aortic stenosis (CAVS) is also one of the common chronic CVD, and its main feature is progressive mineralization of the aortic valve. The research discovered that Ki1642 was given to the IGFII mouse high-fat and high-sucrose (HF-HS) diet for 6 months, and the rate of progression of aortic stenosis was reduced by a factor of three. Since LPAR3 was not expressed in VICs, LPAR1 was suggested to encourage the progress of aortic stenosis. Moreover, studies have shown that the mechanism may be that oxidative transformation of low-density lipoprotein (OxLDL)-LPA promotes the mineralization and osteogenic transformation of VIC by activating the LPAR1-RhoA-NF-κB pathway, thereby improving the development of CAVS 112.

In summary, LPA receptors (especially LPAR1 and LPAR3) play a vital part in the treatment of CVD. LPAR1 and LPAR3 can be used as the next target for the study of cardiovascular drugs.

### LPAR and Fibrosis

Fibrosis is closely associated with end-stage organ failure, leading to severe morbidity and mortality 113. Here we describe the role of LPA and its receptors in the fibrosis progression in order to provide direction for the treatment of disease.

#### LPAR and renal fibrosis

LPA promotes the advancement of renal fibrosis through the LPA_1_ receptor. In the mice with renal interstitial fibrosis (TIF) induced by unilateral ureteral obstruction (UUO), the concentration of ATX and LPA were increased, and real-time reverse transcription polymerase chain reaction (RT-PCR) showed that LPA_1_ receptor was significantly up-regulated and LPA_3_ receptor was significantly down regulated 114. Fibroblast migration and proliferation can be stimulated by ATX-LPA-LPA_1_ receptor signaling 115. The study also found that renal fibrosis was significantly attenuated in LPA_1_^-/-^mice and mice treated with Ki16425. Moreover, when the LPA_1_ receptor was blocked, profibrotic cytokines (connective tissue growth factor and transforming growth factor-β) are significantly down-regulated 114. In one study, it was confirmed that LPA-LPA_1_ signaling directly induces connective tissue growth factor (CTGF) expression in primary proximal tubular epithelial cells via the myocardin-related transcription factor-serum response factor pathway 116. It was worth noting that the glomerular LPA_1_ receptor does not participate in the role of TIF caused by UUO 114. Geng et al. proposed that after renal ischemia-reperfusion injury, LPA mainly induced TGF-β activation through the LPA_2_-Gαq-Rho / ROCK pathway, thereby promoting the development of renal fibrosis 117. Besides, increased expression of LPA_1_ receptor was also detected in TIF induced by a mouse model of nephrotoxic serum (NTS) nephritis, which was more similar to the progression of human kidney disease, and this model is mainly associated with glomerulonephritis 118. Mirzoyan et al. also found that LPA is involved in subtotal nephrectomy (SNx) -mediated renal fibrosis. However, the specific LPA receptors and mechanisms are unclear 119.

#### LPAR and pulmonary fibrosis

Fibroblast accumulation and vascular leakage are principal factors in the pathogenesis of pulmonary fibrosis. In previous studies, LPA levels were remarkably elevated in bronchoalveolar lavage (BAL) fluid collected from patients with idiopathic pulmonary fibrosis (IPF) compared to the normal control group 120,121. In order to study the effect of LPA and LPA receptors in pulmonary fibrosis, bleomycin was used to attack mice lacking LPA_1_. It was found that the excessive accumulation of fibroblasts in damaged lungs and the persistent vascular leakage caused by injury were significantly reduced 121,122. Secondly, AM966, an effective antagonist of LPA_1_ receptor, was used in bleomycin-induced fibrosis model in mice. It not only dwindled vascular leakage, tissue damage, inflammation, and fibrosis but also dwindled the concentration of several pro-fibrosis and pro-inflammatory cytokines in bronchoalveolar lavage fluid (BALF) 123. In a randomized, double-blind, placebo-controlled clinical trial, the use of the LPA_1_ receptor antagonist BMS-986020 significantly slowed the decline of FVC (forced vital capacity) in patients with IPF, and alleviated clinical symptoms 124. It can be seen from the above studies, LPA contributes to the progress of pulmonary fibrosis through the LPA_1_ receptor, especially IPF. In bleomycin-induced pulmonary fibrosis, the progression of fibrosis can also be suppressed by knocking out other LPA receptors. Another study showed that bleomycin-induced lung injury, fibrosis and death can also be alleviated by knocking out the LPA_2_ receptor. Moreover, these phenomena and knockdown of LPA_2_ attenuated LPA-induced expression of transforming growth factor β1 (TGF-β1) and differentiation of lung fibroblasts. Hence LPA_2_ receptor was also important in the process of pulmonary fibrosis 125. Pulmonary fibrosis is a common and serious complication of radiation therapy for lung cancer. LPA_1_/LPA_3_ inhibitor VPC12249 can inhibit the expression of fibroblast-promoting cytokines TGFβ1 and CTGF in vivo, leading to a decrease of fibroblast proliferation and the slow progress of radiation-induced pulmonary fibrosis in mice 126. Regarding the mechanism of LPA and its receptors promoting pulmonary fibrosis, a study suggested that LPA-LPA_1_ pathway could induce bone marrow-derived mesenchymal stem cells (BMSC) to differentiate into myofibroblasts and promote the secretion of extracellular matrix (ECM), thereby promoting pulmonary fibrosis. Moreover, LPA_1_ antagonist Antalpa1 can inhibit these phenomena. After lung injury, myofibroblasts accumulate and activate, secrete excessive ECM, and eventually form fibrotic foci 127.

#### LPAR and liver fibrosis

Inflammation and other hepatic damaging factors activate hepatic stellate cells, make myofibroblasts accumulate and secrete excessive ECM, and then lead to liver fibrosis. The process of fibrosis is also mentioned earlier in this article. Regarding liver damage, studies have shown that when LPA can increase GSH levels, sustained phosphorylation of c-Jun N-terminal kinase (JNK) caused by acetaminophen (APAP) administration is blocked. Furthermore, blocking the production of inflammatory cytokines (TNF-α and IL-1β) protects against APAP-induced acute liver injury. But this process was independent of LPA receptors 128. Hepatitis was a key factor in the development of liver fibrosis. Make use of vitro hepatocytes and in vitro liver culture systems, as well as human liver chimeric mice and HCC tissues, the researchers demonstrated that the ATX-LPA signal axis activates PI3K and stabilizes HIF-1 α, which positively regulates hepatitis C virus (HCV) RNA replication, and this process may be related to LPA1 and LPA3 receptors, promoting disease progression to liver fibrosis and hepatocellular carcinoma 129. In another study, Silymarin, caffeine and their combinations significantly improved liver fibrosis induced by thioacetamide (TAA) by downregulating LPAR1, and downregulated α-SMA, CTGF and TGF-β1, suggesting that LPAR1 may promote the progress of liver fibrosis through α-SMA, CTGF and TGF-β1 130. This is also consistent with the mechanism of pulmonary fibrosis mentioned above. In addition, hepatitis C virus infection promotes liver fibrosis, and when ATX and LPA signals are suppressed, it reduces hepatitis C virus replication 129. The expression of serum ATX was also closely related to liver fibrosis, which also suggests that the ATX-LPA axis plays an important role in liver injury 131.

#### LPAR and other fibrosis

In addition to renal, pulmonary and liver fibrosis, studies have shown that LPA_1_ receptors can also promote the development of dermal fibrosis in bleomycin-induced skin fibrosis models, and is also related to the expression of TGF-β1 and CTGF 132. In a study on ventricular remodeling, it was found that OGN (osteoglycin) in the heart can bind to LPAR3 and attenuate the activation of EGFR signals through the Gα12/13/Rho/ROCK pathway, thereby inhibiting myocardial fibroblast (CMF) proliferation And migration. This suggests that LPAR3 may have a role in regulating fibrosis of the heart 133. LPA and its receptors are also involved in adipose tissue fibrosis. Rancoule et al. injected intraperitoneal injection of Ki16425 (LPAR1/LPAR3 antagonist) to db/db mice to reduce AT (adipose tissue) fibrosis, and experimentally found that LPAR1 may be the main receptor for LPA-promoting fibrosis 134.

### LPAR and diabetes

#### LPAR and diabetes

Diabetes has become a disease that cannot be neglected. The occurrence of diabetes was related to many factors. Here we mainly discuss the effects of LPA and its receptors on diabetes. Current research indicates that LPA and its receptors have different regulatory effects on diabetes and its complications. Studies have shown that LPA boosts glucose uptake by stimulating skeletal muscle and fat cells, thereby reducing blood glucose levels in diabetic mice treated by streptozotocin (STZ). Moreover, the effect of LPA on glucose uptake can be entirely inhibited by Ki16425 (LPA_1/3_ receptor antagonist) 135. LPA also modulated NHE3 through an insulin-independent pathway that improves diarrhea in diabetic patients 136. From the above studies, it can be seen that LPA and its receptors can effectively balance blood sugar and improve some diabetic complications, but in other studies, they have found their effects to be opposite.

#### LPAR and diabetic nephropathy

Diabetic nephropathy is a common complication of diabetes. Its occurrence was related to the angiogenesis and fibrosis promoted by protease, growth factor, cytokines and chemokines released from renal cells 137,138. Studies have found that after using LPA_1/3_ receptor antagonist Ki16425 or BMS002 in db/db mice, kidney damage was significantly reduced, and the progression of diabetic nephropathy was slowed down. This phenomenon was associated with a decrease in GSK3b (Ser9) phosphorylation and SREBP1 (sterol-regulatory element binding proteins 1) activation caused by inhibition of LPAR1, and a subsequent diminish in TGF-β expression. As mentioned earlier in this paper, LPA can induce TGF-β to promote the development of renal fibrosis 137. Beyond that, it may also be related to the decrease in GFR caused by LPAR inhibition 138,139. Since Ki16425 and BMS002 are antagonists of the LPA_1_ receptor and the LPA_3_ receptor, another study used the LPAR1-specific antagonist AM095 to research the function of the LPAR1 in diabetic nephropathy. The study found that in a mouse model induced by STZ, AM095 inhibited renal cell inflammatory signaling cascade and reduced renal damage by inhibiting the TLR4/NF-κB and NADPH oxidase systems. The role of LPA_1_ receptor in the development of diabetic nephropathy was more clearly defined 140.

#### LPAR and obesity

As we all known, obesity is one of the risk factors for diabetes. It was found that after six weeks of the LPA1/3 receptor antagonist Ki16425, the fat mass and white fat cell size of HFHS-fed C57B16 mice increased significantly. Moreover, the ATX-LPA axis can cause obesity-induced insulin resistance by impairing PPARγ expression and activity. The above content showed that LPA receptors played a crucial part in the development of obesity and diabetes 141. After LPAR1-KO mice were fed high-fat and low-fat diets, compared with wild-type mice, no significant changes in body weight and fat were observed. It was suggested that LPAR1 may play a key role in lipid uptake 142.

Current studies on the relationship between diabetes and LPA and its receptors are limited to LPA and LPAR1 and LPAR3. It is still unclear whether other LPA receptors affect the development of diabetes and its complications. Future studies can focus on related drug studies and other receptor effects targeting LPAR1 and LPAR3.

### LPAR and other diseases

#### LPAR and rheumatoid arthritis

Rheumatoid arthritis (RA) is a common chronic inflammatory disease, which is characterized by synovial proliferation, fibroblast-like synoviocytes (FLS) proliferation, angiogenesis, inflammatory cell infiltration and bone destruction of various joints. Miyabe et al. demonstrated that the expression of LPAR1 in the synovium of RA patients was high, and in LPA_1_^-/-^mice or mice using LPA_1_ antagonists, inflammatory cell infiltration and joint destruction in bones were reduced. In vitro experiments it was also shown that LPA_1_ can promote the formation of osteoclasts 143. In the K/BxN serum metastatic arthritis model, after LPA_1/3_ receptor antagonists were used, apoptosis increased, inflammatory mediators decreased, and bone remodeling protein decreased. These ultimately weaken the severity of arthritis. It was found that the LPA-LPA_1_ signaling pathway promoted the development of rheumatoid arthritis by enhancing the proliferation and migration of RA FLS and the production of inflammatory mediators 144, 145. Orosa et al. also confirmed that LPAR1 and LPAR2 were mainly expressed in RA FLS, and when LPAR1 was inhibited, tumor necrosis factor (TNF)-induced FLS proliferation was also reduced 146. Another study suggested that LPA-LPAR1 was involved in SF-stimulated hBMSC migration in RA patients 147.

#### LPAR and Sjogren's syndrome

Sjogren's syndrome (SS) is a chronic autoimmune disease that has not yet been developed to cure it, but studies have shown that treatment with the LPAR1/3 antagonist Ki16425 improves spontaneous development in SS and nonobese diabetic (NOD) mice in adoptive transfer models. SS. Tip LPA and its receptor may be valid targets for SS therapy 148.

#### LPAR and asthma

Asthma is one of the most commonplace lung diseases, and acute bronchoconstriction is one of the main reasons for hospitalization and sudden death from asthma. In animal models, LPA was found to trigger acute allergen and bradykinin-mediated bronchoconstriction by activating carotid body TRPV1 and LPA receptors, causing acute asthma symptoms. It was worth noting that this mechanism has not yet been confirmed in the human body, which can be used as a new research direction 149. In a previous study, a mouse model of allergic asthma with LPA_2_ deficiency was found to be more severe than wild-type mice with pulmonary and systemic inflammation 150. In another study, the role of LPAR2 in suppressing inflammation in asthma was also confirmed. When LPAR2 agonists were used, the inflammation of the lungs and airways of mice caused by allergen sensitization and challenge by HDM (dermatophagoides pteronyssinus) was significantly reduced. This may be related to reduced chemokine production and inhibition of cell migration 151.

#### LPAR and liver regeneration

The role of LPA and its receptors is essential in the overall changing process of liver disease. First, LPA can support the involvement of Rho-kinase by regulating cell morphology and attachment to the extracellular matrix, to reinforce the remodeling of collagen matrix by hepatic stellate cells (HSCs) 152. Second, the more severe the liver damage, the higher the concentration of LPA in plasma 153. Liver disease was a common digestive system disease, and severe liver diseases such as liver cancer have a poor prognosis. Liver resection or liver transplantation was an effective method for treating HCC. Using an enzyme-linked immunosorbent assay (ELISA), it was found that after partial hepatectomy (PHx), circulating LPA increased significantly after 72 hours, and LPAR1 increased in two stages of 12-24 hours and 48 hours-7 days, respectively. The LPAR1-stained cells are HSCs. Both LPAR3 mRNA and LPAR6 mRNA expression increased within 12 hours, but LPAR6 mRNA expression also increased significantly during the subsequent 48 hours to 7 day period. Moreover, LPAR6 was more widely stained than LPAR1 and LPAR3. These results suggest that LPA receptors (LPAR1, LPAR3, LPAR6) can promote liver regeneration after hepatectomy 128.

## Perspectives and future directions

The growth factor-like lipid medium LPA acts as an effective signaling molecule that affects many physiological and pathological processes. So far, research on LPA receptors has progressed in many aspects, especially the most comprehensive study of LPA_1_ receptors, which of course was related to the broader expression of LPA_1_ receptors than other receptors. However, studies on non-EDG receptors, especially LPA_6_ receptors, are not comprehensive. It can be used as one of the future research directions. LPA signaling was associated with pathological responses, including conduction of neuropathic pain, promotion of tumorigenesis and metastasis, promotion of fibrosis, and promotion of atherosclerosis. The six G-protein coupled receptors that bind to it have become clear targets for drug development. Some drugs have entered clinical stages, such as the LPA_1_ receptor antagonists ONO-7300243 and ONO-0300302 are used to treat benign prostatic hyperplasia and inhibit LPA-induced increases in intraurethral pressure in rats and dogs. The LPA_1_ receptor antagonist BMS986020 for idiopathic fibrosis has entered phase II clinical trials but has been discontinued due to noticeable side effects. The LPA_1_ receptor antagonist Debio-0716 can reduce distant metastasis and lymph node metastasis in breast cancer. The LPA_1_ receptor antagonist ONO-8430506 can lower tumor resistance and enhance azithromycin-induced tumor cell death. LPA_1/3_ receptor antagonist SAR100842 can treat systemic sclerosis 65,154. In view of the wide-ranging role of LPA and its receptors in diseases, future research on drugs targeting LPA and its receptors will be of paramount importance.

## Figures and Tables

**Table 1:** The role of LPAR in diseases

LPAR	Disease	Impact	Mechanism	Reference
LPAR1	Ovarian cancer	Aggravate	PI3K/AKT pathway↑	Cui et al.,2019
	Glioblastoma	Aggravate	PKCα↑	Valdés-Rives et al.,2019
	Lung cancer	Aggravate	P53↓	Murph et al.,2007
	Neuropathic pain	Aggravate	Rho-ROCK pathway↑, PKCγ↑,Caα2δ1↑	Inoue et al.,2004
	Neuropathic pain	Aggravate	macrophages/microglia↑	Velasco et al.,2017
	Atherosclerosis	Aggravate	CXCL12 X, HIF-1α↑, SPC↑	Subramanian et al.,2010
	Calcified aortic stenosis	Aggravate	RhoA-NF-κB pathway↑	Nsaibia et al.,2017
	Renal fibrosis	Aggravate	the myocardin-related transcription factor-serum response factor pathway↑, CTGF↑	Sakai et al.,2017
	Pulmonary fibrosis	Aggravate	BMSC to differentiate into myofibroblasts↑, ECM↑	Tang et al.,2014
	Liver fibrosis	Aggravate	α-SMA↑, CTGF↑, TGF-β1↑	Eraky et al.,2018
	Diabetic nephropathy	Aggravate	GSK3b (Ser9) phosphorylation↑, SREBP1↑, TGF-β↑	Li et al.,2017
	Diabetic nephropathy	Aggravate	TLR4 /NF-κB pathway↑, NADPH↑	Lee et al.,2019
	Rheumatoid arthritis	Aggravate	RA FLS↑	Miyabe et al.,2014
LPAR2	Ovarian cancer	Aggravate	Gαi-Src-EGFR-ERK pathway↑	Jeong et al.,2008
	Colon cancer	Aggravate	cPKC↑, β-catenin↑	Yang et al.,2005
	Colon cancer	Attenuate	MAGI-3↑	Lee et al.,2011
	Colon cancer	Aggravate	NHERF-2↑	Lee et al.,2011
	Pancreatic cancer	Attenuate	Gα12/13-Rho pathway↑	Komachi et al.,2009
	Lung cancer	Aggravate	P53↓	Murph et al.,2007
	Atherosclerosis	Aggravate	CXCL12↑,HIF-1α↑, SPC↑	Subramanian et al.,2010
	Renal fibrosis	Aggravate	Gαq-Rho/ROCK pathway↑, TGF-β↑	Geng et al.,2012
	Pulmonary fibrosis	Aggravate	TGF-β1↑	Huang et al.,2013
LPAR3	Ovarian cancer	Attenuate	PI3K/Akt pathway ↑	Li et al.,2019
	Colon cancer	Aggravate	cPKC↑,β-catenin↑	Yang et al.,2005
	Liver cancer	Aggravate	Gαi-ERK-MAPK pathway↑	Zuckerman et al.,2016
	Lung cancer	Aggravate	P53↓	Murph et al.,2007
	Neuropathic pain	Aggravate	macrophages/microglia↑	Velasco et al.,2017
	Atherosclerosis	Aggravate	CXCL12↑, HIF-1α↑, SPC↑	Subramanian et al.,2010
	Cardiac fibrosis	Attenuate	α-SMA↑, CTGF↑, TGF-β1↑	Eraky et al.,2018
LPAR4	-	-	-	-
LPAR5	Melanoma	Attenuate	cAMP↓	Jongsma et al.,2011
	Neuropathic pain	Aggravate	PKD↑,microglia↑	Velasco et al.,2017; Plastira et al.,2017
LPAR6	Liver cancer	Aggravate	pim-3↑	Mazzocca et al.,2015
	Hepatic encephalopathy	Aggravate	Gα12/13-Rho pathway↑, BBB↑	Herr et al.,2019; Masago et al.,2018
LPAR	Ovarian cancer	Aggravate	Rac-NOXROS-HIF1α pathway↑	Ha et al.,2018
	Alzheimer's disease	Aggravate	Aβ accumulation↑, tau hyperphosphorylation↑, neuronal dysfunction↑	Ramesh et al.,2018

PKCα: protein kinase Cα; PKCγ: protein kinase Cγ; LPAR: lysophosphatidic acid receptors; CXCL12: CXC motif ligand 12; HIF-1α: hypoxia-inducible factor-1α; SPC: smooth muscle progenitor cells; CTGF: connective tissue growth factor; BMSC: bone marrow-derived mesenchymal stem cells; ECM: extracellular matrix; SREBP1: sterol-regulatory element binding proteins 1; TGF-β1: transforming growth factor β1; NADPH: nicotinamide adenine dinucleotide phosphate; RA: rheumatoid arthritis; ERK: extracellular regulated protein kinases; cPKC: classical protein kinase C; MAGI-3: inverted orientation-3; NHERF-2: Na^+^/H^+^ exchange regulatory factor-2; MAPK: mitogen-activated protein kinase; cAMP: cyclic adenosine monophosphate; PKD: protein kinase D; BBB: blood-brain barrier.

## Abbreviations

LPA: lysophosphatidic acid
GPCRs: G-protein coupled receptors
LPAR: lysophosphatidic acid receptor
EDG: endothelial differentiation gene
ECL: extracellular loop
ICL: intracellular loop
VZ: ventricular zone
TMIII: transmembrane-spanning III
TMV: transmembrane-spanning V
TMVII: transmembrane-spanning VII
VIC: valve interstitial cell
PDZ: PSD-95/DlgA/ZO-1
NHERF: Na^+^/H^+^ exchange regulatory factor
MAGI-2: inverted orientation-2
MAGI-3: inverted orientation-3
HCC: hepatocellular carcinoma
PI3K: phosphatidylinositol 3-kinase
PKCd: protein kinase Cd
STAT3: signal transducers and activators of transcription 3 (STAT3)
PRG: PDZ-RhoGEF
LARG: leukemia-associated Rho GEF
SPC: smooth muscle progenitor cells
CPSP: central post-stroke pain
COX-2: cyclooxygenase-2
PGI2: prostacyclin
cAMP: cyclic adenosine monophosphate
YAP: Yes-associated protein
TAZ: transcriptional co-activator with PDZ-binding motif
TCR: T cell antigen receptor
ERK: extracellular regulated protein kinases
DRG: dorsal root ganglia
NHE3: Na^+^/H^+^Exchanger 3
PLD: phospholipase D
TRPA1: transient receptor potential ankyrin 1
TRPV1: transient receptor potential vanilloid 1
VEGF: vascular endothelial growth factor
IL: interleukin
EGFR: epidermal growth factor receptor
OSC: ovarian serous cystadenocarcinoma
ITH: intratumoral heterogeneity
BC: breast cancer
ATX: autotaxin
MAPK: mitogen-activated protein kinase
RNAi: RNA interference
cPKC: classical protein kinase C
NERF-2: Na^+^/H^+^ exchange regulatory factor
PLC: phospholipase C
CARMA3: caspase recruit domain and MAGUK domain containing 3
MALT1: mucosa-associated lymphoid tissue 1
NF-κB: nuclear factor-kappa κB
PKCδ: protein kinase Cδ
GBM: Glioblastoma
PIT: photochemically induced thrombosis
CNS: central nervous system
PKD: protein kinase D
NP: neuropathic pain
CPZ: cuprizone
MS: multiple sclerosis
FH: fetal hydrocephalus
PHH: posthemorrhagic hydrocephalus
NPC: neural progenitor cells
BBB: blood-brain barrier
TCA: tricyclic antidepressant amitriptyline
AD: alzheimer's disease
CVD: cardiovascular disease
SMCs: smooth muscle cells
CXCL12: CXC motif ligand 12
HIF: hypoxia-inducible factor
DGPP: dioctylglycerol pyrophosphate
PA: dioctylphosphatidic acid
ADP: adenosine diphosphate
CAVS: calcified aortic stenosis
HF-HS: high-fat and high-sucrose
UUO: unilateral ureteral obstruction
TIF: ubulointerstitial fibrosis
RT: reverse transcription
PCR: polymerase chain reaction
CTGF: connective tissue growth factor
NTS: nephrotoxic serum
OxLDL: oxidative transformation of low-density lipoprotein
BAL: bronchoalveolar lavage
IPF: idiopathic pulmonary fibrosis
BALF: bronchoalveolar lavage fluid
FVC: forced vital capacity
TGF: transforming growth factor
BMSC: bone marrow-derived mesenchymal stem cells
ECM: extracellular matrix
JNK: c-Jun N-terminal kinase
APAP: acetaminophen
HCV: hepatitis C virus
TAA: thioacetamide
STZ: streptozotocin
PPARγ: peroxisome proliferator-activated receptor γ
SS: Sjogren's syndrome
RA: Rheumatoid arthritis
FLS: fibroblast-like synoviocytes
NOD: nonobese diabetic
HSCs: hepatic stellate cells
ELISA: enzyme-linked immunosorbent assay
PHx: partial hepatectomy
